# Bioinformatics analysis and experimental verification of Notch signalling pathway-related miRNA–mRNA subnetwork in extracellular vesicles during *Echinococcus granulosus* encystation

**DOI:** 10.1186/s13071-022-05391-8

**Published:** 2022-07-30

**Authors:** Jian Gao, Xuan Zhou, Ling Liu, Guodong Lv, Qiulian Hou, Xiaofan Zhang, Yujuan Shen

**Affiliations:** 1 National Institute of Parasitic Diseases, Chinese Center for Disease Control and Prevention (Chinese Center for Tropical Diseases Research); NHC Key Laboratory of Parasite and Vector Biology; WHO Collaborating Centre for Tropical Diseases; National Center for International Research on Tropical Diseases, Shanghai, 200025 China; 2 College of Basic Medicine, Xinjiang Medical University, Urumqi, 830011 China; 3 Clinical Laboratory, Tumor Hospital Affiliated to Xinjiang Medical University, Urumqi, 830011 China; 4 Clinical Medical Research Institute, The First Affiliated Hospital of Xinjiang Medical University, Urumqi, 830011 China; 5 Department of Laboratory Medicine, Sun Yat-Sen Memorial Hospital, Sun Yat-Sen University, Guangzhou, China; 6 School of Basic Medical Sciences and Forensic Medicine, North Sichuan Medical College, Nanchong, China

**Keywords:** *Echinococcus granulosus*, Protoscolex, Extracellular vesicles, miRNA, Encystation, Notch signalling pathway

## Abstract

**Background:**

Encystation of the protoscoleces (PSCs) of *Echinococcus granulosus* is the main cause of secondary hydatid dissemination in the intermediate host. Extracellular vesicles (EVs) can transfer miRNAs into parasite cells to regulate mRNA expression. However, loading of developmental pathway-related miRNAs, such as those related to the Notch signalling pathway in EVs is unclear. Thus, we screened the miRNA-mRNA subnetwork involved in the Notch pathway during *E. granulosus* encystation in vitro and assessed changes in expression in the parasite and EVs.

**Methods:**

mRNAs and miRNAs differentially expressed (DE) between PSCs and microcysts (MCs) were screened using high-throughput sequencing. DE mRNAs obtained from transcriptome analysis were intersected with mRNAs predicted to be targets of the conserved DE miRNAs of a small RNA library. DE miRNA functions were analysed using public databases, and a miRNA–mRNA subnetwork related to the Notch pathway was established. Notch pathway-related mRNA and miRNA expression of worms and EVs at different times was verified.

**Results:**

In total, 1445 DE mRNAs between MCs and PSCs were screened after the intersection between 1586 DE mRNAs from the transcriptome and 9439 target mRNAs predicted using 39 DE miRNAs from the small RNA library. The DE mRNAs were clustered into 94 metabolic pathways, including the Notch pathway. Five DE miRNAs, including the most significantly expressed new DE miRNA, egr-new-mir0694-3p, corresponding to four target mRNAs (EgrG_000892700, EgrG_001029400, EgrG_001081400 and EgrG_000465800) were all enriched in the Notch pathway. The expression of the above mRNAs and miRNAs was consistent with the results of high-throughput sequencing, and the expression of each miRNA in EVs was verified. Annotated as ADAM17/TACE in the Notch pathway, EgrG_000892700 was down-regulated during PSC encystation. egr-miR-4989-3p and egr-miR-277a-3p expression in EVs after encystation was nearly five times that in EVs before encystation, which might regulate the expression of EgrG_000892700.

**Conclusions:**

Five miRNAs corresponding to four target mRNAs may be involved in regulating the Notch pathway during the PSC encystation. EVs may regulate the expression of EgrG_000892700 in PSCs because of continuous targeting of egr-miR-4989-3p and egr-miR-277a-3p and participate in the regulation the Notch pathway. The study might expand new ideas for blocking the secondary infection of *E. granulosus* PSCs via EVs miRNAs.

**Graphical Abstract:**

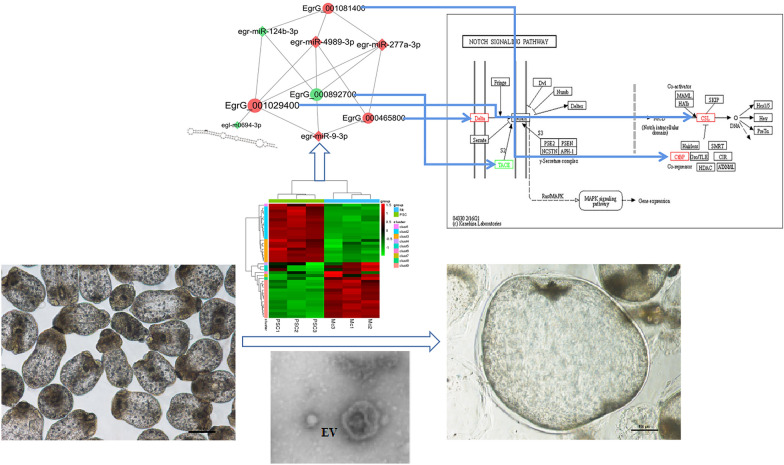

**Supplementary Information:**

The online version contains supplementary material available at 10.1186/s13071-022-05391-8.

## Background

Cystic echinococcosis (CE), a zoonotic disease caused by *Echinococcus granulosus* larvae, poses a serious threat to human health and the socio-economy worldwide [1]. Approximately 5−30% of the population in western China is positive for the *E. granulosus* antibody, indicating that many individuals have been exposed to this parasite [2], highlighting the urgency of prevention and treatment.

During the pathogenic stage of CE, hydatid, large amounts of hydatid fluid (HF), daughter cysts and protoscoleces (PSCs) are in the cyst wall. As PSCs can differentiate into new hydatid cysts in intermediate hosts such as humans, the dissemination and secondary infection by PSCs after the rupture of the cyst wall is the main reason for postoperative recurrence of the disease. Although the life cycle of *E. granulosus* is well studied, the main developmental mechanism remains unclear, especially the mechanism via which non-coding RNAs regulate worm development.

miRNAs are endogenous non-coding RNAs 22 nucleotides in length [3], which affect the molecular mechanism of various diseases [4]. Since 2011, when Cuche et al. [5] first cloned 38 different *E. granulosus* miRNAs, and 2020, when Bai et al. [6] identified 167 known mature miRNAs or miRNA stars, high-throughput sequencing has considerably boosted research on miRNAs. Studies have shown that various known and newly discovered *E. granulosus* miRNAs potentially interact with five major signalling pathways, Wnt, cAMP, Hedgehog, NF-κB and Notch [7], confirming that miRNAs play an important regulatory role in the differentiation and development of *E. granulosus*. Notch signal transduction plays a key role in cell development and cell-cell communication in vertebrates and invertebrates, including the regulation of cell fate, migration, differentiation and proliferation [8]. Studies related to the development and regulation of *E. granulosus* have mostly focused on the Wnt pathway and rarely on the Notch signalling pathway, indicating that further investigations are required.

Extracellular vesicles (EVs) encapsulated by lipid bilayers are released during eukaryote growth and development, play an important role in the cellular microenvironment and are rich in miRNAs and mRNAs [9]. Siles-Lucas et al. [10] reported an exocrine body in the cyst fluid of sheep affected with liver echinococcosis for the first time in 2017. Zhang et al. [11] identified miRNAs which may be related to host immunity and pathogenesis in the culture supernatant of PSCs and cyst fluid exocrine body of *E. granulosus*. These studies suggested that EVs can act as carriers of miRNAs and may play a key role in different developmental stages, which warrants further investigation.

The purpose of this study was to screen the mRNAs and miRNAs involved in the regulation of the Notch signalling pathway during *E. granulosus* encystation and to obtain preliminary information regarding the EVs harbouring miRNAs that participate in the regulation of *E. granulosus* encystation. This study provides a reference for developing new ideas for blocking the secondary infection of *E. granulosus* PSCs via EVs miRNAs.

## Methods

### Parasite material and in vitro culture of *E. granulosus* PSCs

Liver hydatid cysts were collected aseptically from *E. granulosus*-infected Xinjiang Altay sheep in a slaughterhouse in Urumqi, China. The HF and PSCs were obtained from the fertile cysts under aseptic conditions and were placed in 50-ml Falcon tubes. The PSCs were washed 5–8 times using 0.9% NaCl containing 100 U/ml penicillin and 100 μg/ml streptomycin (Gibco/Life Technologies, Carlsbad, CA, USA). The PSCs were purified using aseptic 40- and 80-mesh stainless steel screens, followed by digestion with 1% aseptic pepsin solution of pH 2–3 for 30 min in a 37 ℃ water bath. The digested PSCs were washed ten times with normal saline containing 1% penicillin-streptomycin and finally re-suspended in complete Roswell Park Memorial Institute (RPMI)-1640 medium (Gibco/Life Technologies) containing 200 U/ml penicillin and 200 μg/ml streptomycin, 4 mg/ml glucose and 5% fetal bovine serum (Gibco/Life Technologies). The fetal bovine serum was ultracentrifuged at 110,000 × *g* overnight to remove EVs. Viability was determined using an eosin stain dye exclusion test. The qualified PSCs (survival rate ≥ 90%) re-suspended in complete RPMI-1640 medium were inoculated in T25 culture bottles at 2000 cells/ml and cultured in a 5% CO_2_ constant temperature incubator (Thermo Scientific Forma™ 310, Waltham, MA, USA) set at 37 ℃. The medium was changed every 2–3 days to establish a 90-day in vitro culture model of *E. granulosus* PSCs, and the morphological changes of PSCs during encystation were recorded under an inverted microscope digital imaging system (Nikon ECLIPSE Ts2R, Nikon, Japan). The encystation rate and change in cyst diameters were calculated after every 5 days, and the change curve was drawn. Average microencystation rate was calculated as (microcysts/total number of worms in the field of view) × 100%. Ten PSCs in the field of view were randomly selected, the diameters of the PSCs or MCs were measured using a microscope micrometre, and the average diameter of the three fields of view was calculated.

### Library preparation and RNA-seq analysis

PSCs and MCs cultured in vitro for > 60 days were collected separately. The samples were added to 1 ml TRIzol reagent (Invitrogen, Frederick, MD, USA), mixed thoroughly and stored at − 80 °C. Total RNA was extracted using the TaKaRa MiniBEST Universal RNA extraction kit (TaKaRa, Japan). The mRNA-seq library (non-strand-specific) was constructed and sequenced by Personal Biotech Co., Ltd. (Nanjing, China). The quality, quantity and integrity of the total RNAs were assessed using Thermo Scientific NanoDrop 2000 and an Agilent 2100 Bioanalyser (Agilent Technologies, Santa Clara, CA, USA).

The mRNAs with polyA structure were enriched in the total RNA using the oligo (dT) magnetic bead method and disrupted into fragments of about 300 bp in length using ion interruption to facilitate the generation of clusters in the on-machine sequencing process. Using the mRNAs as template, the first strand of cDNA was synthesised using a six-base random primer and reverse transcriptase, and the second strand cDNA was synthesised using the first strand cDNA as the template. After constructing the library, the library fragments were enriched using polymerase chain reaction (PCR), and a library of 450-bp fragments was selected. The quality, total concentration and effective concentration of the library were detected using the Agilent 2100 Bioanalyser. The libraries containing different index sequences were further mixed in proportion. The mixed library was uniformly diluted to 2 nM, and the single-stranded library was formed via alkali denaturation. Finally, using next-generation sequencing based on the Illumina sequencing platform, paired-end sequencing was performed on these libraries.

After the 3′ ends with the linker sequence and low-quality data were removed from the mRNA raw data (raw data), the high-quality sequences were compared with the *E. granulosus* reference genome (https://www.ncbi.nlm.nih.gov/genome/10706) using HISAT2. Fragments per kilobase of transcript per million mapped reads were obtained using HTSeq from the read count of the aligned sequence number. DESeq was used to obtain the standardised average expression of mRNAs (baseMean) such that the expression levels of different genes were comparable. The DE mRNAs were screened using DE Seq (threshold: |log2FoldChange|> 1 and *P* < 0.05).

### Small RNA library construction, sequence analysis and miRNA identification

Small RNA was extracted using RNAiso for small RNA kit (TaKaRa). The small RNA library was constructed and sequenced by Personal Biotech Co., Ltd., and was established using NEB Next multiplex small RNA library prep set for Illumina kit (New England Biolabs Inc., Ipswich, MA, USA). Using total RNA as the raw material, small RNAs were efficiently enriched via ligation of 3′-end and 5′-end joints. The first strand cDNA was synthesised via reverse transcription, and the library fragments were enriched using PCR. The PCR amplification products were recovered and purified via gel electrophoresis, and their quality was assessed using the Agilent 2100 Bioanalyser and Agilent high-sensitivity DNA kit. The library was quantified based on fluorescence using the Quant-iT PicoGreen dsDNA assay kit (Invitrogen, Carlsbad, CA, USA). The effective library concentration was quantified using the StepOnePlus real-time PCR systems (Thermo Scientific). Multi-sample DNA libraries (multiplexed DNA libraries) were homogenised and mixed in equal volumes. The mixed library was gradually diluted and quantified and then sequenced in PE150 mode on the Illumina sequencing platform.

The miRNA raw data were saved in FASTQ format. High-quality data (clean reads) with sequence length of 18−36 nucleotides were counted, and duplicate sequences were removed to obtain unique reads. The reference sequence of the miRNA precursor and mature body of *E. granulosus* were obtained from the miRBase database. The comparison between unique reads and the *E. granulosus* genome sequence, annotation of the obtained miRNAs and identification of conserved miRNAs were performed using Bowtie. For unannotated sequences, unknown miRNAs were obtained by predicting new miRNA precursors using mireap. The number of sequences aligned to a gene (read count) was counted based on the number of sequences aligned to mature miRNAs of *E. granulosus*. In cases where miRNAs with the same name were present in different locations, the abundance of the miRNA that appears for the first time was used for subsequent analysis, and the new miRNA sequence and expression level were predicted simultaneously. The read count and the exon count per million mapped reads were calculated using DESeq (threshold: fold change ≥ 2.0 and *P* < 0.05). Considering the 3′ untranslated region of the mRNAs of *E. granulosus* as the target sequence, the target mRNAs of the miRNAs differentially expressed between PSCs and MCs were predicted using miRanda.

### Bioinformatics analysis

The DE mRNAs between PSCs and MCs in the transcriptome were intersected with the target mRNAs predicted using the miRNAs differentially expressed between PSCs and MCs in the small RNA library to screen the final target mRNAs. Gene Ontology (GO) and Kyoto Encyclopaedia of Genes and Genomes (KEGG) enrichment analyses of target mRNAs were performed using topGO and KAAS, respectively, and the hypergeometric distribution method. *P* < 0.05 was considered significant for target mRNA enrichment. Significant GO terms were found, pathways to which target mRNAs been enriched were determined, and miRNA biological functions related to target mRNAs were annotated. The target mRNAs enriched in the Notch signalling pathway were screened, and the subnetwork of miRNAs-mRNAs related to the Notch signalling pathway was drawn using Cytoscape. According to the results of high-throughput sequencing, miRNAs with ≥ 3 connections to the target mRNAs (degree) met the candidate criteria.

### Transmission electron microscopy, nanoparticle tracking analysis and Western blotting for EV identification

EVs were collected from the culture supernatant on days 3−10, 55−62 and 80−87 of culture, which correspond to the three time points of encystation of PSCs in vitro. Before collection, worms were washed 3−5 times with sterile phosphate-buffered saline (PBS) and resuspended in serum-free medium. The parasite culture medium was harvested and changed every 12 h. The PSCs culture media were successively centrifuged at 300 × *g* for 10 min, 2000 × *g* for 20 min and finally 10,000 × *g* for 40 min to remove large dead cells and cell debris. The supernatants were filtered using low-protein binding 0.22 μm pore filters (Millipore, Bedford, MA, USA), concentrated using Amicon Ultra-15 100 kDa ultrafiltration tube (Millipore), and centrifuged at 4 °C, 4000 × *g* for 10 − 20 min to collect the concentrated liquid. The concentrated liquids were ultracentrifuged at 110,000 × *g* for 90 min at 4 °C to pellet the vesicles using an Optima L-100 XP ultracentrifuge (Beckman Coulter, Indianapolis, IN, USA); the pellets were resuspended in 100 μl PBS and stored at − 80 °C until use.

Twenty microlitres of the EV suspension was dropped on the copper mesh of the electron microscope, dried under an incandescent lamp, negatively stained using 2% uranyl acetate aqueous solution for 5 min, washed with pure water to remove excess dye solution and dried using filter paper. After drying at 25 °C, the sample was observed under a transmission electron microscope (G2 Spirit BioTWIN, Tecnai, Germany).

For nanoparticle tracking analysis, the EV suspension was sufficiently mixed via pipetting and diluted 1000-fold. Before the samples were detected on the machine, the cuvette of the nanoparticle tracking analyser (ZetaView PMX 110, Particle Metrix, Meerbusch, Germany) was pre-washed with deionised water more than three times and calibrated using polystyrene microspheres (110 nm). The detection test results were processed using ZetaView 8.04.02 SP2 (Particle Metrix).

For western blotting, the EV samples were lysed on ice using radioimmunoprecipitation assay lysis buffer (Abcam, USA) containing 1% phenylmethylsulfonyl fluoride for 30 min, and then centrifuged at 10,000 × *g*, 4 °C, for 10 min. Protein sample concentrations were determined using the bicinchoninic acid assay. The EV pellet (20 µl) was mixed with 10 µl of a solution containing 5 × sodium dodecyl sulphate and 20 µl 1 × PBS and then boiled at 100 °C for 10 min. After returning to 25 °C, the protein samples were centrifuged briefly and processed for western blotting per standard procedures. Primary antibodies against CD63 (1:1000) (Abcam) and enolase (1:1000) (Cell Signaling Technology, Danvers, MA, USA) were used. For all secondary antibody incubations, 1:5000 dilution of horseradish peroxidase-conjugated or goat anti-rabbit antibodies (Abcam) were used. The membranes were visualised using the Gel Doc XR + imaging system (Bio-Rad, USA).

### Quantitative reverse transcription-PCR (qRT-PCR)

Worm samples were collected on days 3, 55 and 80 of in vitro culture and were established as three experimental groups: PSC, MC and HC. Total RNA and small RNA were extracted from PSCs and MCs as mentioned before for high-throughput sequencing. The process for extracting total RNA from EVs included an additional step of mixing 10 μl nucleic acid precipitation acryl carrier (Solarbio, China) in isopropanol. The purity and concentration of nucleic acid samples were detected using the nucleic acid protein analyser Nanodrop 1000 (Thermo Scientific). *U6* and *GAPDH* were used as internal reference genes. The primers were designed using Primer Premier 5.0 and synthesised using Sangon Biotech Co., Ltd. (Shanghai, China). The sequences of the primers are shown in Table 1. The qRT-PCR results were verified using the Mir-X miRNA first-strand synthesis kit and the Mir-X miRNA qRT-PCR TB Green® kit (Clontech, Takara, Japan) using a real-time fluorescent quantitative PCR instrument (Roche LightCycler480 II, USA). The reaction conditions were as follows: 95 ℃ for 3 min, 1 cycle; 95 °C for 5 s and 60 °C for 45 cycles. Two replicate wells were used for each sample, the number of cycles (Ct value) at which the fluorescence signal reached the threshold was recorded, and the target genes were quantified using the 2^−ΔΔCt^ method. Statistical analysis and graphing were performed using GraphPad Prism 8.0.2 software. The statistical methods were *t* test and one-way ANOVA-Turkey's multiple comparison test. Regarding the critical value q for Tukey's multiple comparisons, the study chose to compare each mean with the other means, dividing the difference between the means being compared by the standard error of the difference and calling the quotient *q*.

**Table 1:** Primer sequence

Gene	Primer 5′ to 3′	
egr-miR-4989-3p	RT	CTCAACTGGTGTCGTGGAGTCGGCAATTCAGTTGAGTCTCAGAT
	F	ACACTCCAGCTGGGAAAATGCACCAACT
egr-miR-124b-3p	RT	CTCAACTGGTGTCGTGGAGTCGGCAATTCAGTTGAGGGTATTC
	F	ACACTCCAGCTGGGTAAGGCACGCGGT
egr-miR-9-3p	RT	CTCAACTGGTGTCGTGGAGTCGGCAATTCAGTTGAGTTTGTTTG
	F	ACACTCCAGCTGGGCAAGGCTAGATTTC
egr-new-mir0694-3p	RT	CTCAACTGGTGTCGTGGAGTCGGCAATTCAGTTGAGTACATAC
	F	ACACTCCAGCTGGGTGGAATGTTGTGAA
Universal primer	R	TGGTGTCGTGGAGTCG
U6	F	CAGAATGTCGCCGTTGTTG
	R	TGGGATTTAAGGGCTCTGC
EgrG_000892700	F	CGGCATTCTGGCTCTTACTT
	R	AAGCCCGTGTTCGTGTTG
EgrG_001029400	F	AATGCCTCGGACTTCTTCG
	R	ACCGCCTTTGATGTTGGAT
EgrG_001081400	F	GGAATAGTTGGTCTCGGTCGTA
	R	CAATGCCGTCGGATAGGTAG
EgrG_000465800	F	TCAGGATGACGATGAAGATGC
	R	GCTGTGCTAACGGAAGTGGA
GAPDH	F	TGAAGATGTACCGTCTAGTGCC
	R	TTTCTCATCCTTACCAATCGTCT

*RT* specific reverse transcription primers, *F* forward primers, *R* reverse primers

## Results

### Morphological observation of the cystic model of *E. granulosus* PSCs in vitro

**Fig. 1:**
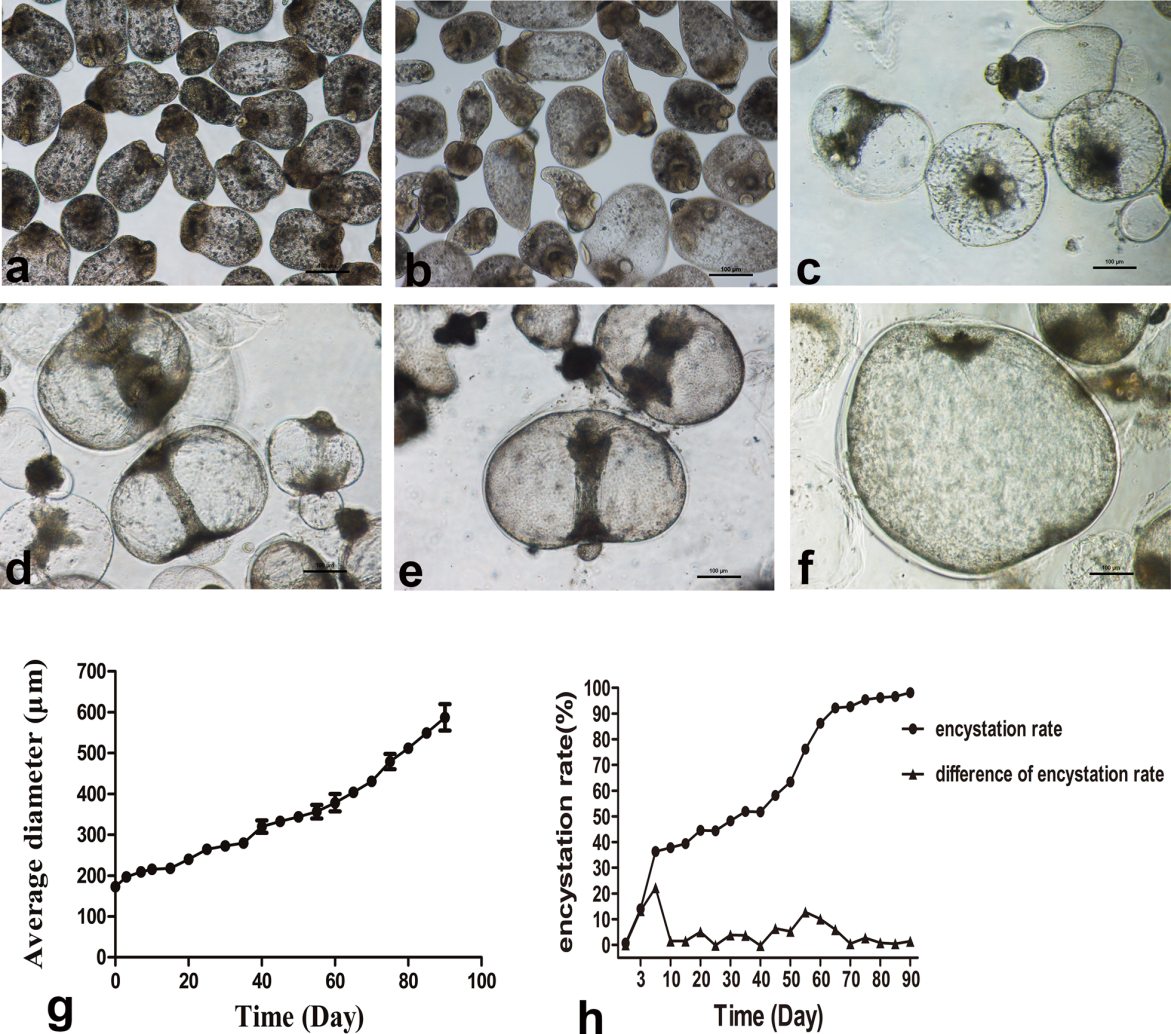
An in vitro encystation model of PSCs of *E. granulosus*. **a**–**f** Morphological changes of PSCs after 3, 7, 20, 40, 60 and 80 days of culture in vitro. Scale bar: 100 μm. **g** Average diameter of PSCs. **h** The encystation rate of PSCs. *PSCs* protoscoleces, *MCs* microcysts

The initial PSCs presented irregular elliptical bodies 180−220 μm in diameter. Most of the scolexes were inverted, and the body contained a large number of highly refractive calcium particles. The active worms showed strong refraction and slight peristalsis under the microscope, while the dead worms were shrunken and smaller than the live ones, the internal structures of which were blurred. With prolongation of in vitro culture time, more scolexes turned out, the worms became larger, and they gradually vacuolated. Calcium particles, suckers, hooks and other structures disappeared, and the cuticle layer on the surface of the body wall usually appeared after 20 days of culture in vitro. Two peaks of encystation were observed on days 7 and 55 after the PSCs were cultured in vitro. After 80 days of in vitro culture, the encystation rate of the worms reached 96.19%, the average diameter of the MCs was 512.03 ± 12.89 μm, and the maximum diameter was 846.33 μm. The laminated layer of the MCs had thickened and the cysts were highly transparent (Fig. 1).

### Functional annotation of the target mRNAs predicted from the miRNAs differentially expressed between PSCs and MCs

**Fig. 2:**
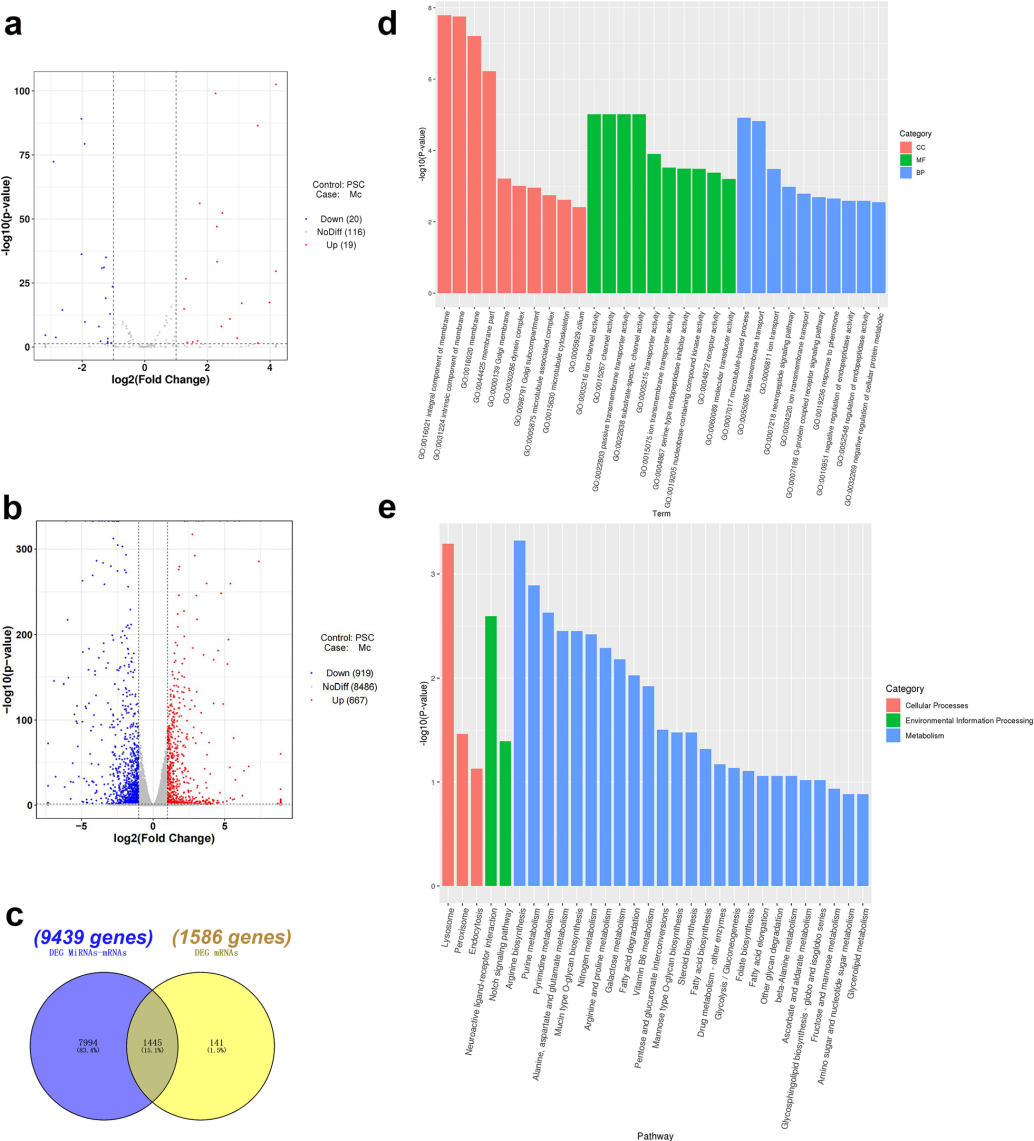
Bioinformatics analysis of DE miRNAs and mRNAs (PSCs vs MCs). **a** Volcan of DE miRNAs. **b** Volcan of DE mRNAs. **c** Venn diagram based on the intersection between the target mRNAs predicted by DE miRNAs and mRNAs. **d** GO analysis of target mRNAs between PSCs and MCs (Top 10 of terms). *CC* cellular component, *MF* molecular function, *BP* biological process. **e** KEGG analysis of target DE mRNAs between PSCs and MCs. *PSCs* protoscoleces, *MCs* microcysts, *DE* differentially expressed

After the analysis of the miRNA library, 39 conserved miRNAs with differential expression between PSCs and MCs were screened out. Compared to that in PSCs, 19 miRNAs were upregulated and 20 were downregulated in MCs (Additional file 1: Table S1). Furthermore, 9439 target mRNAs were predicted (Fig. 2a). In the transcriptome library, 1586 mRNAs with differences between PSCs and MCs were screened out. Among them, 667 mRNAs were upregulated, and 919 mRNAs were downregulated in MCs (Fig. 2b). After the 1586 DE mRNAs of the transcriptome were intersected with 9439 target mRNAs predicted using the DE miRNAs, 1445 target mRNAs were screened out (Fig. 2c).

In total, 1430 GO terms were generated from 1445 target mRNAs using GO enrichment analysis. The three categories of GO terms are cellular component (CC), molecular function (MF) and biological process (BP). GO enrichment entries from each GO category show the top ten entries (Fig. 2d). Eighteen entries with *P* < 0.05 and FDR (corrected value of *P* value) < 0.05 were selected from the above 30 entries (Additional file 2: Table S2).

 GO enrichment analysis revealed that the target mRNAs related to transport channel activity, transmembrane transport protein activity and molecular functions of other proteins and enzymes in the MF category accounted for 55.56% of the total mRNAs. The target mRNAs related to the CC category accounted for 27.78% of the total, such as the occurrence of membranes. The target mRNAs related to microtubule formation and ion transport across membranes in the BP category accounted for 16.67% of the total mRNAs.

To assess the role of developmental-related signalling pathways in PSC encystation, the gene function clusters of target mRNAs were further analysed using the KEGG database. In total, 1445 target mRNAs were clustered into 94 metabolic pathways, which included cellular process (CP), environmental information processing (EP) and metabolism (M). The top 30 pathways with the lowest *P* in KEGG classification were selected and displayed in the form of a bar graph (Fig. 2e). Twenty-five of the top 30 pathways of target mRNA enrichment were related to metabolism such as the amino acid or nucleotide metabolic pathways. The Notch signalling pathway was among the only two EP-related pathways, which was also at the 17th place in the list of the most significantly (*P* < 0.05) enriched pathways.

### Prediction and screening of differential new miRNAs during the encystation of *E. granulosus* PSCs

Fifty new miRNAs were screened after matching the following three conditions: the precursor secondary structure was a classic stem-loop structure (egr-new-mir0694 as an example, Additional file 3: Fig. S1), minimum folding free energy (MFE) <  − 20 kcal/mol and minimum folding freedom energy index (MFEI) > 0.85. Six new miRNAs, expression of which differed between PSCs and MCs, were screened out (Additional file 4: Table S3). Compared to that in PSCs, five new miRNAs were downregulated, and one miRNA was upregulated in MCs. egr-new-mir0694-3p showed the highest expression level among the 50 new miRNAs and the downregulated new miRNAs. Hence, its role in the encystation of PSCs warrants further investigation.

### Interactions among miRNA–mRNA in the Notch signalling pathway

**Fig. 3:**
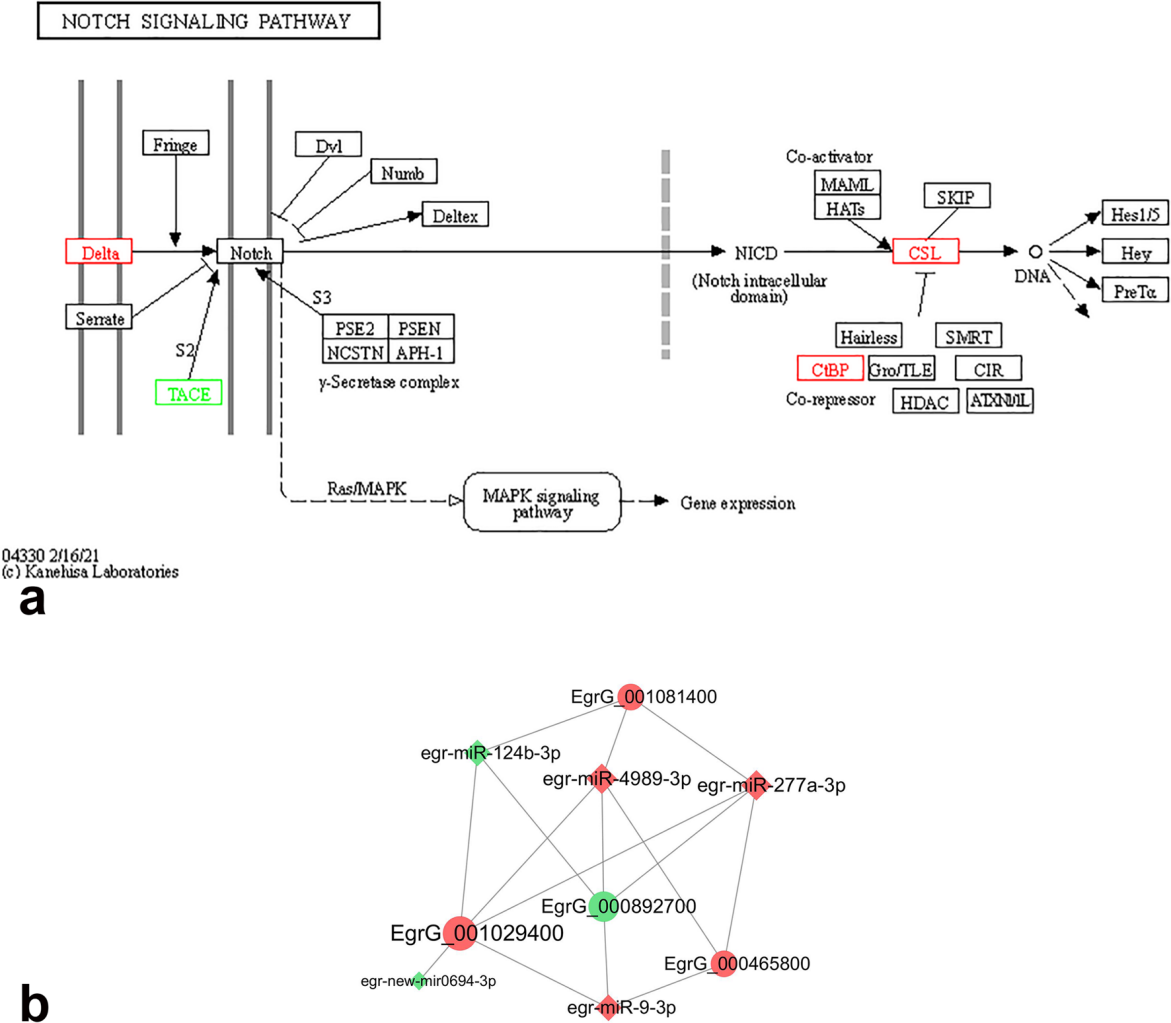
Interactions among miRNA and mRNA in the Notch signalling pathway. **a** Notch signalling pathway. Red box: genes upregulated in MCs compared to PSCs; green box: genes downregulated in MCs compared to PSCs; PSCs, protoscoleces; MCs, microcysts. **b** The miRNA–mRNA subnetwork related to Notch signalling pathway. Circle: target mRNA; diamond: miRNA; red: upregulation of expression; green: downregulation of expression; the larger the graph area, the more genes associated with this gene

In the KEGG enrichment analysis, four target mRNAs predicted by the conserved DE miRNAs were enriched in the Notch signalling pathway. The target mRNAs were annotated using the Swiss-Prot database (Table 2). Furthermore, 37 conserved miRNAs related to these four target mRNAs were found. With the number of connections with the target mRNAs (degree) ≥ 3 as the screening condition, a miRNA–mRNA subnetwork of four conserved differential miRNAs corresponding to four target mRNAs was formed (Fig. 3). This has been predicted to be related to EgrG_001029400. The new miRNA, egr-new-mir0694-3p, with the highest differential expression, was also enriched in the miRNA–mRNA subnetwork.

**Table 2:** Annotation of target mRNAs related to notch signalling pathway

Id	Expression*	Protein annotation	Notch signalling pathway annotation
EgrG_001029400	Up	Suppressor of hairless protein, SUH	CSL
EgrG_001081400	Up	C-terminal Binding Protein, CtBP	CtBP
EgrG_000465800	Up	Delta-like ligand 4, DLL4	Delta
EgrG_000892700	Down	Adisintegrin and metalloproteinase17, ADAM17	TACE

*Compared with PSCs

### Identification of EVs from PSCs

**Fig. 4:**
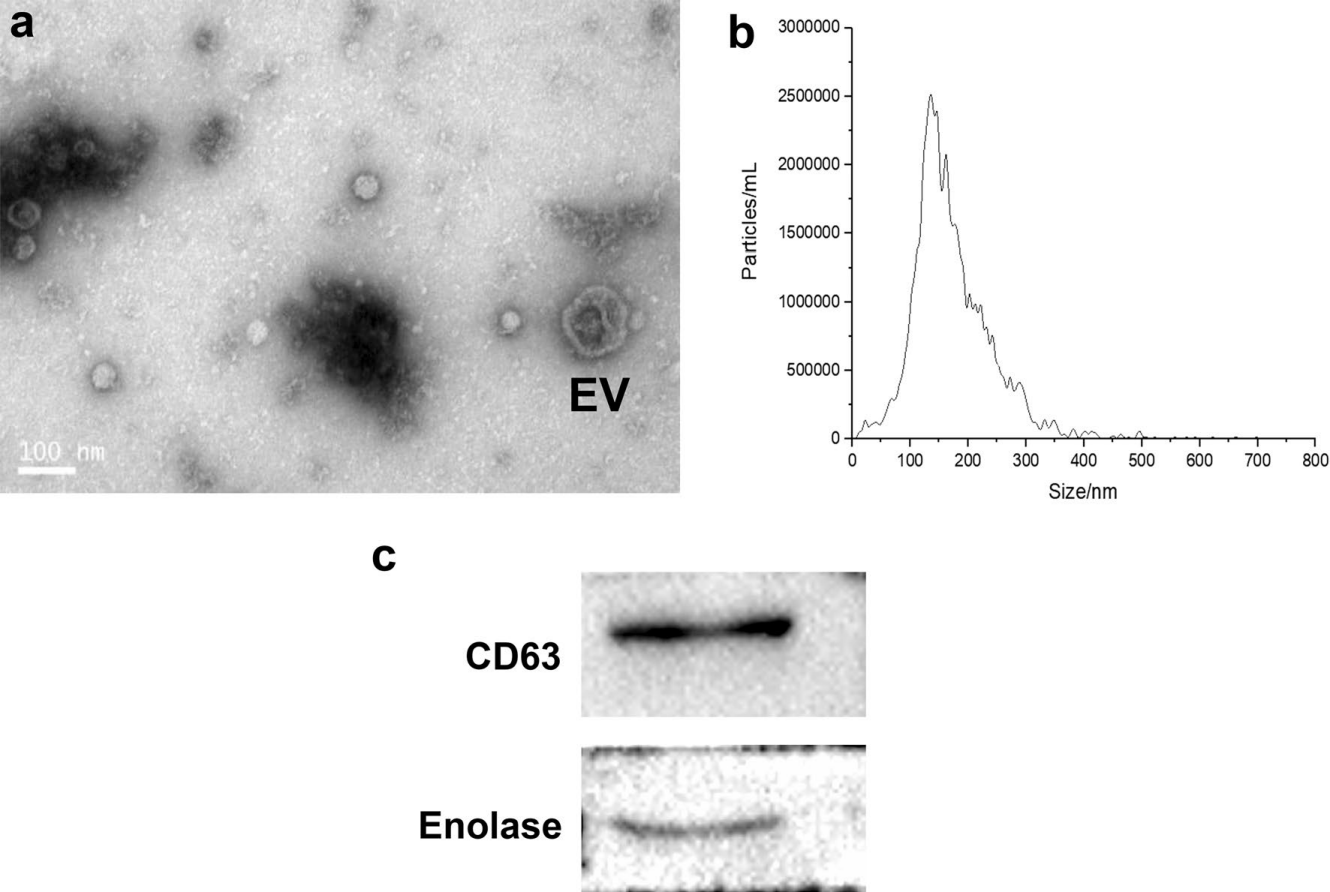
Identifications of EVs from PSCs. **a** Images of the rounded or cup-shaped vesicles obtained using negative staining under transmission electron microscopy. Scale bar: 100 nm. **b** The diameter distribution analysis of the purified EVs assessed using nanoparticle tracking analysis. **c** EVs markers CD63 and enolase were measured using Western blotting analysis. *EVs* extracellular vesicles, *PSCs* protoscoleces

Transmission electron microscopy identified cup-shaped vesicles, which was characteristic of EVs (Fig. 4a). Particle size measurements obtained using nanoparticle tracking analysis showed that most of the purified vesicles derived from PSCs were 40–150 nm in diameter (Fig. 4b). The expression of CD63 and enolase, markers of EVs, was verified using western blotting (Fig. 4c).

### qRT-PCR validation of the Notch signalling pathway-related miRNA–mRNA subnetwork

**Fig. 5:**
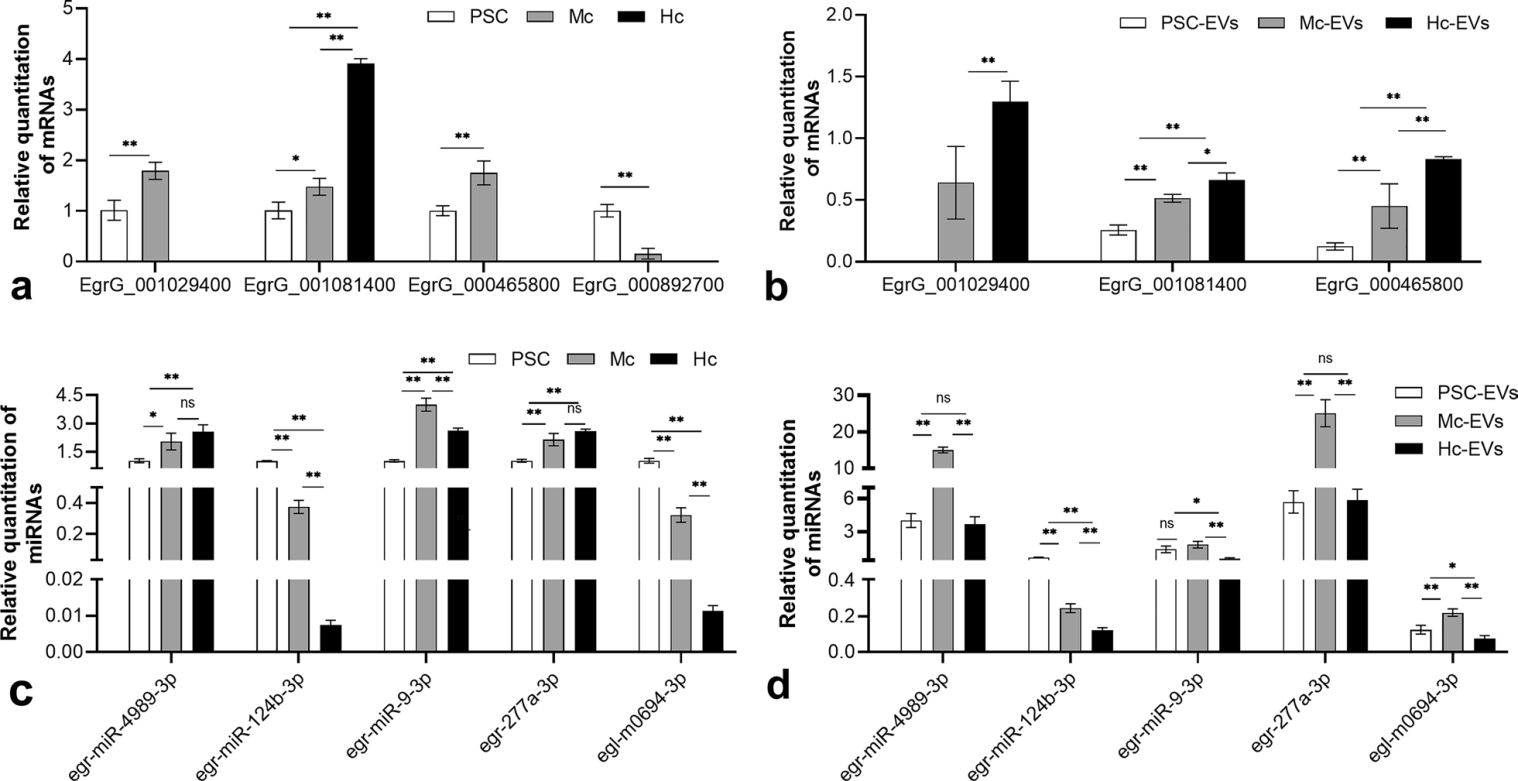
Relative quantification of the target mRNAs and candidate miRNAs of Notch signalling pathway. **a** Relative quantification of the target mRNAs in the worms. **b** Relative quantification of the target mRNAs in the EVs. **c** Relative quantification of the candidate miRNAs in the worms. **d** Relative quantification of the candidate miRNAs in the EVs. *PSC* protoscoleces, *MC* microcyst after 55 days cultured in vitro, *HC* microcyst after 80 days cultured in vitro; —: differences between adjacent groups; ——: difference between the first group and the last group. **P < *0.01*; *P < *0.05*; ns: *P* > *0.05*. *EVs* extracellular vesicles

The trends in the expression of Notch signalling pathway-related mRNAs validated using qRT-PCR were consistent with the results of high-throughput sequencing. Compared to that observed in the PSC group, EgrG_001081400 (Turkey test, *P* = 0.018, *q* = 5.561, df = 6), EgrG_000465800 (*t* test, *P* = 0.007, *t* = 5.084, df = 4) and EgrG_001029400 (*t* test, *P* = 0.007, *t* = 5.184, df = 4) target mRNAs in MC group were upregulated, while EgrG_000892700 was downregulated (*t* test, *P* = 0.001, *t* = 9.137, df = 4). The expression of EgrG_001081400 was significantly higher in the HC than MC group (Turkey test, *P* = 0.000, *q* = 28.95, df = 6), and the expression levels of the other three mRNAs were below the lower limit of detection using PCR (Fig. 5a). Except for EgrG_001029400, which was not expressed in the PSC EVs, the expression of the three upregulated mRNAs was verified at the three time points of EV collection, and changes in expression were identical to those observed in the worm (Fig. 5b).

miRNA-mRNA subnetwork analysis results were predicted and further verified using qRT-PCR. egr-miR-4989-3p, egr-miR-9-3p and egr-miR-277a-3p might exert regulatory effects on EgrG_000892700; egr-miR-124b-3p and egr-new-mir0694-3p might exert regulatory effects on EgrG_001029400; egr-miR-124b-3p might exert regulatory effect on EgrG_001081400. Except for the slight decrease in egr-miR-9-3p expression in the HC group, the expression levels of egr-miR-4989-3p, egr-miR-9-3p and egr-miR-277a-3p showed an upward trend in the worms during encystation, while egr-miR-124b-3p and egr-new-mir0694-3p showed a downward trend. These results were consistent with those of high-throughput sequencing (Fig. 5c).

Egr-miR-4989-3p (Turkey test, *P* = 0.000, *q* = 28.25, df = 6), egr-miR-277a-3p (Turkey test, *P* = 0.0001, *q* = 14.72, df = 6) and egr-new-mir0694-3p (Turkey test, *P* = 0.0003, *q* = 12.11, df = 6) expression in EVs from the MC group was higher than that from the HC group (that of egr-miR-4989-3p and egr-miR-277a-3p was almost five times higher), while no significant difference in the expression of the above two miRNAs was observed between the EVs of the PSC and HC groups (Turkey test, *P* = 0.8485, *q* = 0.7830, df = 6; Turkey test, *P* = 0.9955, *q* = 0.1278, df = 6). egr-miR-9-3p expression did not differ significantly between EVs from the PSC and MC groups (Turkey test, *P* = 0.1789, *q* = 2.913, df = 6), but was slightly lower in EVs from HC group (Turkey test, *P* = 0.0020, *q* = 8.753, df = 6) (Fig. 5d).

## Discussion

Although surgery and imidazole drugs are the main methods for treating CE, blocking of encystation and development of PSCs in the host are critical for avoiding secondary infections and postoperative recurrence. Therefore, the highly plastic bi-directional development of PSCs is the focus of research on the developmental mechanism of *E. granulosus* [12]. Protoscolex is an important stage of development in the complex life cycle of *E. granulosus*, which can develop into adults in the gastrointestinal tract of the terminal host and into echinococcosis cyst in the liver and lung of the intermediate host. Even when cultured in MEM basal medium without additional components such as serum for 15 days, the in vitro encystation rate of PSCs might reach 35.65% [13], indicating that developmental regulation is still the main mechanism underlying morphological changes of the parasite. Microcysts of *E. granulosus* cultured > 60 days in vitro may significantly improve the modelling rate of the intraperitoneal inoculation infection model [14, 15]. MCs were more active than PSCs in increasing interleukin (IL)-6 levels to promote the chronic infection process of hydatid and downregulate IFN-γ levels to promote the survival of worms [16], suggesting that MCs might play a key role in secondary hydatid infections. We speculated whether the Notch signalling pathway played any major regulatory role in *E. granulosus* PSCs encystation and whether EVs participated in the regulation of *E. granulosus* encystation by targeting non-coding RNAs related to developmental pathway. The above-mentioned hypotheses must be urgently tested.

MicroRNA is a key regulator of worm-host interaction[17, 18]. The transcriptional regulation of miRNAs is one of the key mechanisms for controlling animal and plant development, as they participate in the post-transcriptional regulation of almost all cellular signalling pathways in animals and plants and are widely expressed in *E. granulosus*, *E. multilocularis* and *E. canadensis* [19, 20]. Both Bai et al. [21] and Cucher et al. [5] reported that miR-2, miR-71 and miR-125 were the most highly expressed miRNAs among the 76 known miRNAs of *E. granulosus* (s.s.). In addition, miRNAs were found to exhibit tissue- and phase-specific expression. MiR-277, let-7, miR-71, miR-10, miR-2 and miR-9 were specifically expressed in the cyst walls of secondary hydatid cysts and PSCs of G1 and G7 genotype, whereas miR-125 was only detected in PSCs and pre-microcysts. Additionally, three miRNAs (let-7, miR-71 and miR-2) were highly expressed in PSCs of metacestodes (cyst walls), suggesting that their expression was developmentally regulated [22]. GO enrichment analysis revealed that the DE miRNAs in *E. granulosus* and their potential targets may participate in nutrient metabolism and bi-directional development of the nervous system [23]. By analysing the mechanism via which miRNAs regulate cystic development in *E. granulosus*, the target that controls the encystation of *Echinococcus* and triggers CE can be identified [24].

High-throughput sequencing technology has boosted research on non-coding RNAs of *Echinococcus*, and the mapping of the miRNA–mRNA subnetwork has improved the accuracy of predicting gene function in the corresponding regulatory pathways [25]. The establishment of the bi-directional development model of PSCs in vitro to adults and MCs and the difference in observation period often lead to different conclusions. We found two peaks of encystation on the 7th and 55th days after the PSCs were cultured in vitro. Bai et al. [6] used RNA and small RNA sequencing to characterise the mRNAs and miRNAs expression at 0–24 h and 7–14 days during the bi-directional development of PSCs. In total, 963 mRNAs and 31 miRNAs were differentially expressed in the early development of PSCs to adult worms, whereas 972 mRNAs and 27 miRNAs were differentially expressed in the early development of PSCs to MCs. Pairwise comparison between the two developmental patterns showed that 172 mRNAs and 15 miRNAs were differentially expressed at three time points. The expression of most of these genes changed temporally at 24 h or 7 days. GO enrichment analysis revealed that the DEGs involved in early adult worm development were associated with nervous system development and carbohydrate metabolism, whereas the DEGs involved in early cystic development were associated with transmembrane transporter and nucleoside triphosphatase activities. In our study, the GO enrichment analysis showed that 55.56% of the target mRNAs were related to the transport channel activity, transmembrane transport protein activity and molecular functions of other proteins and enzymes in the MF category. These conclusions are partially consistent with those of the previous reports.

Fan et al. [26] also established an in vitro encystation model of up to 80 days and screened 32,401 transcripts and 14,903 cDNAs, which revealed several new genes and transcripts, stage-specific genes and DE genes involved in encystation in an in vitro culture cycle separated by 20 days. Function-enriched 1991 DEGs showed that 1094, 148 and 263 DEGs belonged to the CC, BP and MF GO categories, respectively, of which 7090 and 122 genes were significantly differentially expressed. More than half of the DEGs belonged to the CC category, which included integral components of the membrane, intrinsic components of the membrane and membrane parts.

After KEGG analysis, 1445 DE mRNAs between PSCs and MCs were clustered into 94 metabolic pathways, which included CP, EP and M categories. Among them, 25 of the top 30 pathways with the most significant enrichment were associated with metabolism. This is consistent with the results of Zheng et al. [27], which showed that *E. granulosus* had 500–550 KEGG terms associated with metabolism and possessed complete pathways for glycolysis, tricarboxylic acid cycle and the pentose phosphate pathway.

*Echinococcus granulosus* possesses several complete signalling pathways, including those for mitogen-activated protein kinase, ErbB, Wnt, Notch, Hedgehog, TGF-β, Jak-Stat and insulin signalling pathways [28]. In proteomic analysis of PSCs [29], among the 1197 proteins expressed by the protocephala, 632 proteins were involved in 276 regulatory pathways, such as the Wnt, Notch, Hedgehog, NF-κB, cAMP and bile acid signalling pathways that were closely related to the development of *E. granulosus*. β-Catenin was mainly distributed in the hooks and dispersed cells of the PSCs. Furthermore, the relative transcription of β-catenin gradually decreased during 0 to 10 days of in vitro culture. The mRNA level of *wnt2* in adult *E. granulosus* was higher than that in PSCs, while that of *wnt4, wnt5, wnt11A* and *wnt11B* in PSCs was higher than that in the adult worms. All six *wnt* gene family members were distributed in the forward region of PSCs [30]. The Wnt signalling pathway may play an important role in the formation of the *E. granulosus* body axis and in the development of germ cells.

In this study, the Notch signalling pathway, instead of the Wnt signalling pathway, was 1 of the 18 pathways with the most significant enrichment in KEGG analysis, and one of the 2 EP-related pathways in the top 30 pathways with the most significant enrichment. The Notch signalling pathway was found in organisms as diverse as worms and humans. The *notch* gene played a critical role in tissue development, regulation of cell proliferation and differentiation and cell fate in all metazoans [31, 32]. Dezaki et al. [33] found that the fold difference of *notch* was significantly higher at the cultivated MCs in vitro and germinal layer of the hydatid cyst than those in the other developmental stages of *E. granulosus*. Although the two pathways were related to each other [34], the Wnt signalling pathway may be more involved in the development of PSCs to adults, while the Notch signalling pathway may be more active during the encystation of *E. granulosus* PSCs.

A miRNA-mRNA subnetwork of five DE miRNAs corresponding to four target mRNAs (TACE, CSL, CtBP and Delta) was formed, which was enriched in members of the Notch signalling pathway. Although three other mRNAs tended to be upregulated during encystation, EgrG_000892700, annotated as ADAM17, was downregulated during this process. ADAM17 can mediate S2 cleavage under special, nonphysiological circumstances. It cleaveed the T-ALL-associated mutant forms of Notch signalling pathway that were ligand-independent in tissue culture cells [35]. ADAM10 was necessary for Notch processing when Notch signalling pathway was activated by a ligand, while ADAM17 was the major protease that processes Notch signalling pathway and was activated independent of the ligand in both flies and mammals [36]. If ADAM17 was downregulated, the nuclear accumulation of NICD (Notch1 intracellular domain, NICD) would be significantly reduced, and transcription and cell proliferation regulated by the Notch signalling pathway would be inhibited, which was corroborated by the fact that PSCs do not proliferate, but encyst in vitro. NICD-mediated constitutive activation of Notch in the developing chick heart promoted differentiation of conduction cells and inhibited that of cardiomyocytes, while inactivation of the Notch signalling pathway by expressing a dominant negative version of Su(H) increased the expression of myocardial lineage markers at the expense of that of the conduction cell markers [37]. Activation and inhibition of the Notch signalling pathway may guide the differentiation outcomes of different cells, and its role in the regulation of *E. granulosus* encystation should be further studyed.

Egr-miR-4989-3p, egr-miR-9-3p and egr-miR-277a-3p may exert regulatory effects on EgrG_000892700, which were verified using PCR. These three miRNAs may be involved in the inhibition of ADAM17 expression, thereby affecting the downstream transcriptional regulation of the Notch signalling pathway. Although the role is not clear, the newly identified miRNA, egr-new-mir0694-3p, was predicted to only be involved in regulation of EgrG_001029400 in the subnetwork, expression of which was significantly down-regulated during encystation. Our conclusions will be more convincing if biosynthetic miRNA mimics, inhibitors or agomir and antagomir are used to increase in vitro co-culture and animal experiments.

This study further focused on the transport of five miRNAs related to the Notch signalling pathway in the EVs of PSCs during encystation. Studies on EVs of worms mainly focused on schistosomiasis. Both the adults and eggs of *Schistosoma japonicum* can secrete EVs [38, 39]. In total, 403 proteins, 15 known *S. japonicum* miRNAs, and 19 new miRNAs were detected in the EVs of *S. japonicum*. The EVs of *S. japonicum* can be internalised by mammalian cells, and the proteins and miRNAs present in them can be transferred to recipient cells to downregulate the expression of the corresponding target genes. Wang et al. [40] found that the EVs of *S. japonicum* contained a special secretory protein, which can mediate the immune activity of M1 type macrophages and increase the secretion of pro-inflammatory cytokines, tumour necrosis factor-α (TNF-α) and IL-12. The reports on EVs of cestodes are currently limited to *Taenia asiatica* [41], *Taenia saginata* and *Echinococcus* [19], and they mainly focused on interactions between EVs of cyst fluid and immune cells such as macrophages and dendritic cells [42, 43]. Yang [44] found one type of 110-KDa EVs in the HF of *E. granulosus* containing 25 miRNAs, which were internalised by sheep peripheral blood mononuclear cells in a time-dependent manner to upregulate IL-10, TNF-α and IRF5, and downregulate IL-1β, IL-17 and CD14. Exosome-related miRNAs contribute to the interaction between host and parasites in worms [45] and affect host immune response [46]. However, reports on the mechanism via which EV-related miRNAs regulate the development of parasite are rare.

In total, 118 miRNAs and 2361 lncRNAs were identified in the PSC EVs of *E. granulosus* [11], the types and numbers of which were more than those in the EVs in the cyst fluid. Among them, egr-miR-4989-3p was the most abundant in EVs from both PSCs and cyst fluid. Zheng [47] confirmed that emu-miR-4989-3p was encapsulated in EVs produced by *E. multilocularis*, which significantly affected the production of nitric oxide in macrophages and the expression of several key components involved in the LPS/TLR4 signalling pathway, suggesting that miR-4989-3p exerts an immunomodulatory effect during *Echinococcus* infection.

Consistent with Zhang et al. [11], egr-bantam-3p, egr-miR-4989-3p and egr-miR-277a-3p were detected in PSC EVs, which were verified using qRT-PCR. egr-bantam-3p was downregulated (data not shown), but egr-miR-4989-3p and egr-miR-277a-3p were upregulated during encystation, and they exerted a potential regulatory effect on the EgrG_000892700 enriched in the Notch signalling pathway. Except for EgrG_001081400 in MCs, the expressions of the four mRNAs enriched in the Notch signalling pathway were lower than the detection limit after 80 days of in vitro culture. Except for EgrG_000892700, all mRNAs were detected in the EVs. In the middle stage of encystation, the expressions of egr-miR-4989-3p and egr-miR-277a-3p in EVs were nearly five times higher than those before the encystation. Since targeting miRNAs to regulate intracellular mRNA expression is one of the biological functions of EVs, this study speculates that EVs may regulate the expression of EgrG_000892700 in worms via continuous targeting of egr-miR-4989-3p and egr-miR-277a-3p, inhibit the transcription of the Notch signalling pathway and participate in the regulation of *E. granulosus* encystation. This conclusion needs to be further confirmed by observing the direct effect of EVs on the *E. granulosus* encystation.

This study verified the association of miRNAs–mRNAs subnetwork related to the Notch signalling pathway, and mechanisms such as the actual regulatory role and upstream–downstream relationship of each gene in this subnetwork on the Notch signalling pathway remain to be explored further.

## Conclusion

In this study, we screened the miRNA-mRNA subnetwork that may be involved in regulating the Notch signalling pathway during the PSCs encystation process using bioinformatics analysis. A miRNA-mRNA subnetwork of five miRNAs corresponding to four target mRNAs enriched in the Notch signalling pathway was formed, which contained a new miRNA, egr-new-mir0694-3p. Furthermore, the changes in the expression of the above mRNAs and miRNAs in the worms and EVs were evaluated. EVs may participate in the regulation of EgrG_000892700 expression in worms through continuous targeting of egr-miR-4989-3p and egr-miR-277a-3p, inhibit transcription of the Notch signalling pathway genes and participate in the regulation of *E. granulosus* encystation. Thus, this study provides a preliminary mechanism via which EVs may regulate the encystation of *E. granulosus* PSCs by targeting and transporting Notch signalling pathway-related miRNAs. After being internalisated by PSCs, the effect and regulation of the targeted delivery of the corresponding miRNAs by EVs during the encystation of worms might be further explored.

## Supplementary Information

**Additional file 1: Table S1.** Conservative DE miRNAs between PSCs and MCs.

**Additional file 2: Table S2.** GO analysis of target DE mRNAs (*P* < 0.05).

**Additional file 3: Figure S1.** Secondary structure of new miRNA egr-new-mir0694 precursor.

**Additional file 4: Table S3.** Six new DE miRNAs between PSCs and MCs.

## Data Availability

The dataset supporting the conclusions of this article is available in the NCBI repository. The unique persistent identifier is BioProject ID: PRJNA832430, and hyperlink to dataset is in https://www.ncbi.nlm.nih.gov/Traces/study/?acc=PRJNA832430.

## Abbreviations

CE: Cystic echinococcosis
EVs: Extracellular vesicles
HF: Hydatid fluid
MCs: Microcysts
PCR: Polymerase chain reaction
PSCs: Protoscoleces
DE: Differentially expressed
