# Supplementary material for: Bioinformatics analysis and experimental verification of Notch signalling pathway-related miRNA–mRNA subnetwork in extracellular vesicles during Echinococcus granulosus encystation
Source: Parasit Vectors. 2022 Jul 30;15:272. doi: 10.1186/s13071-022-05391-8 (PMC9338502; doi:10.1186/s13071-022-05391-8)
Supplement: Supplementary file 1 — Additional file 1: Table S1. Conservative DE miRNAs between PSCs and MCs. [file 13071_2022_5391_MOESM1_ESM.docx]

**Table S1 Conservative DE miRNAs between PSCs and MCs**

| **id** | **Expression^*^** | **basemean** | **log2FoldChange** | ***P-*Value** |
| --- | --- | --- | --- | --- |
| egr-miR-9-5p | Up | 4498924.605 | 3.6058399 | 3.74707E-87 |
| egr-miR-4989-3p | Up | 350179.5597 | 2.3019225 | 9.18045E-48 |
| egr-miR-745-3p | Up | 321765.4244 | 1.2552085 | 1.38822E-15 |
| egr-miR-190-5p | Up | 39455.88449 | 1.311226 | 2.31018E-27 |
| egr-miR-277a-3p | Up | 26038.41881 | 2.4797341 | 4.28922E-53 |
| egr-miR-9-3p | Up | 11663.7998 | 2.2607011 | 9.6263E-100 |
| egr-miR-4989-5p | Up | 1616.215122 | 1.7540806 | 8.39987E-57 |
| egr-miR-31-5p | Up | 233.1949683 | 4.18534 | 2.932E-103 |
| egr-miR-10293-3p | Up | 167.4000474 | 2.3106971 | 4.85792E-34 |
| egr-miR-31-3p | Up | 35.58733562 | 3.9824845 | 4.07891E-18 |
| egr-miR-10256-5p | Up | 35.20842533 | 3.0961448 | 8.30042E-18 |
| egr-miR-10249-3p | Up | 27.62381681 | 2.7159161 | 1.01961E-11 |
| egr-miR-10227a-5p | Up | 24.86525902 | Inf | 2.35965E-30 |
| egr-miR-2d-5p | Up | 22.70304422 | 2.4468508 | 8.62082E-09 |
| egr-miR-10250-5p | Up | 15.03858479 | 1.3552199 | 0.014923507 |
| egr-miR-10229a-5p | Up | 10.85726947 | 1.5320588 | 0.010516398 |
| egr-miR-10233-3p | Up | 9.472685265 | 1.6829258 | 0.003820878 |
| egr-miR-10227e-5p | Up | 6.21888502 | 2.950188 | 0.000306368 |
| egr-miR-10229b-3p | Up | 2.601984138 | 3.6107723 | 0.027096962 |
| egr-miR-7b-5p | Down | 350149.842 | -1.028968 | 2.60492E-24 |
| egr-miR-7-5p | Down | 253031.4958 | -2.020921 | 8.16299E-90 |
| egr-bantam-3p | Down | 224954.3298 | -1.922683 | 4.75633E-80 |
| egr-miR-125-5p | Down | 86145.99022 | -1.36707 | 1.52958E-31 |
| egr-miR-281-3p | Down | 24869.92996 | -1.304459 | 8.44703E-32 |
| egr-miR-124b-3p | Down | 11735.77122 | -1.239648 | 9.55472E-36 |
| egr-miR-3479b-5p | Down | 1140.850684 | -2.915039 | 4.34144E-73 |
| egr-miR-153-3p | Down | 757.6793838 | -1.112242 | 1.22334E-13 |
| egr-bantam-5p | Down | 505.6211807 | -1.247398 | 8.11431E-20 |
| egr-miR-133-5p | Down | 290.2134118 | -2.019615 | 5.62178E-37 |
| egr-miR-1992-3p | Down | 87.21406315 | -1.465889 | 9.46044E-09 |
| egr-miR-10234-5p | Down | 82.26253195 | -2.631287 | 3.41441E-15 |
| egr-miR-10243-5p | Down | 54.42111791 | -1.91383 | 1.62529E-10 |
| egr-miR-219-3p | Down | 36.48777043 | -1.182162 | 0.000482997 |
| egr-miR-7-3p | Down | 25.51186714 | -1.186466 | 0.008253918 |
| egr-miR-10235-5p | Down | 23.17492432 | -1.070468 | 0.023515598 |
| egr-miR-124a-5p | Down | 16.18188473 | -1.415397 | 0.005324637 |
| egr-miR-281-5p | Down | 14.4362523 | -1.185215 | 0.031312757 |
| egr-miR-10234-3p | Down | 12.43880696 | -3.175837 | 2.34158E-05 |
| egr-miR-10235-3p | Down | 9.930642114 | -2.842916 | 0.000179547 |
| **basemean:** the normalized average expression of the overall gene of PSCs and MCs; **log2FoldChange:** the log2 logarithmic value of the multiple of the gene expression difference between PSCs and MCs;  **Inf:** the calculated result is infinitely large or infinitely small and cannot be displayed.  ***:**Compared with PSCs. | | | | |
