# Supplementary material for: Bioinformatics analysis and experimental verification of Notch signalling pathway-related miRNA–mRNA subnetwork in extracellular vesicles during Echinococcus granulosus encystation
Source: Parasit Vectors. 2022 Jul 30;15:272. doi: 10.1186/s13071-022-05391-8 (PMC9338502; doi:10.1186/s13071-022-05391-8)
Supplement: Supplementary file 2 — Additional file 2: Table S2. GO analysis of target DE mRNAs (P < 0.05). [file 13071_2022_5391_MOESM2_ESM.docx]

**Table S2 GO analysis of target DE mRNAs (*P*<0.05)**

| **Category** | **Term** | **GO ID** |
| --- | --- | --- |
| CC | integral component of membrane | GO:0016021;  GO:0031224 |
| CC | membrane | GO:0016020 |
| CC | membrane part | GO:0044425 |
| CC | Golgi membrane | GO:0000139 |
| MF | ion channel activity | GO:0005216 |
| MF | channel activity | GO:0015267 |
| MF | passive trans membrane transporter activity | GO:0022803 |
| MF | substrate-specific channel activity | GO:0022838 |
| MF | transporter activity | GO:0005215 |
| MF | ion transmembrane transporter activity | GO:0015075 |
| MF | serine-type endopeptidase inhibitor activity | GO:0004867 |
| MF | nucleobase-containing compound kinase activity | GO:0019205 |
| MF | receptor activity | GO:0004872 |
| MF | molecular transducer activity | GO:0060089 |
| BP | microtubule-based process | GO:0007017 |
| BP | transmembrane transport | GO:0055085 |
| BP | ion transport | GO:0006811 |
| CC, cellular component; MF, molecular function; BP, biological process. | | |
