# Supplementary figures and images for: Bioinformatics analysis and experimental verification of Notch signalling pathway-related miRNA–mRNA subnetwork in extracellular vesicles during Echinococcus granulosus encystation
Source: Parasit Vectors. 2022 Jul 30;15:272. doi: 10.1186/s13071-022-05391-8 (PMC9338502; doi:10.1186/s13071-022-05391-8)

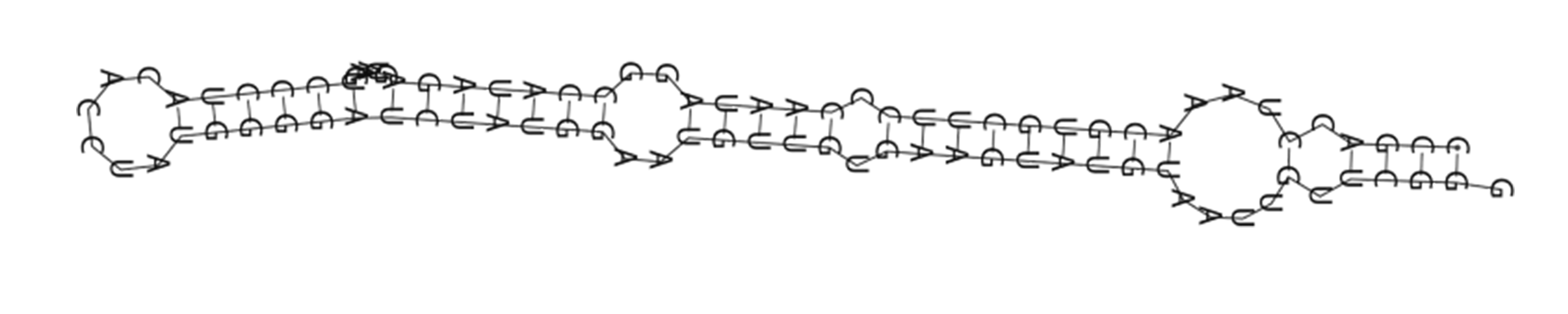

Supplement: Supplementary file 3 — Additional file 3: Figure S1. Secondary structure of new miRNA egr-new-mir0694 precursor. [file 13071_2022_5391_MOESM3_ESM.tif]
