# Supplementary material for: Bioinformatics analysis and experimental verification of Notch signalling pathway-related miRNA–mRNA subnetwork in extracellular vesicles during Echinococcus granulosus encystation
Source: Parasit Vectors. 2022 Jul 30;15:272. doi: 10.1186/s13071-022-05391-8 (PMC9338502; doi:10.1186/s13071-022-05391-8)
Supplement: Supplementary file 4 — Additional file 4: Table S3. Six new DE miRNAs between PSCs and MCs. [file 13071_2022_5391_MOESM4_ESM.docx]

**Table S3 Six new DE miRNAs**

| id | Expression^*^ | baseMean | log2FoldChange | *P*-value |
| --- | --- | --- | --- | --- |
| egr-new-mir0694-3p | DOWN | 1044715 | -1.272161 | 0.0006846 |
| egr-new-mir0755-3p | DOWN | 214.6396 | -1.843311 | 0.0003181 |
| egr-new-mir0024-5p | UP | 20.719225 | 2.0352186 | 0.0017998 |
| egr-new-mir0517-3p | DOWN | 18.840022 | -1.318678 | 0.045803 |
| egr-new-mir0712-5p | DOWN | 5.2830587 | -3.669683 | 0.0114489 |
| egr-new-mir0219-3p | DOWN | 2.188075 | -Inf | 0.0492107 |
| **basemean:** the normalized average expression of the overall gene of PSCs and MCs; **log2FoldChange:** the log2 logarithmic value of the multiple of the gene expression difference between PSCs and MCs;  **Inf:** the calculated result is infinitely large or infinitely small and cannot be displayed.  *:Compared with PSCs. | | | | |
